# Microstructure and superconducting properties of high-rate PLD-derived GdBa_2_Cu_3_O_7−*δ*_ coated conductors with BaSnO_3_ and BaZrO_3_ pinning centers

**DOI:** 10.1038/s41598-019-51348-w

**Published:** 2019-10-23

**Authors:** Alexey V. Ovcharov, Pavel N. Degtyarenko, Vsevolod N. Chepikov, Alexander L. Vasiliev, Sergey Yu. Gavrilkin, Igor A. Karateev, Alexey Yu. Tsvetkov, Andrey R. Kaul

**Affiliations:** 1 National Research Center “Kurchatov Institute”, Moscow, 123182 Russia; 2 SuperOx, Moscow, 117246 Russia; 3 Joint Institute for High Temperature of Russian Academy of Sciences, Moscow, 125412 Russia; 4 Lomonosov Moscow State University, Moscow, 119991 Russia; 5 Shubnikov Institute of Crystallography of Russian Academy of Sciences, Moscow, 117333 Russia; 6 Moscow Institute of Physics and Technology (State University), Dolgoprudny, Moscow Region 141701 Russia; 7 P.N. Lebedev Physical Institute of the Russian Academy of Sciences, Moscow, 119991 Russia

**Keywords:** Superconducting properties and materials, Surfaces, interfaces and thin films

## Abstract

The microstructure of GdBa_2_Cu_3_O_7−*δ*_ based on superconducting tapes with BaSnO_3_ and BaZrO_3_ artificial pinning centers formed by high-rate pulse laser deposition in SuperOx Japan was studied by scanning/transmission electron microscopy. The artificial pinning centers have adopted columnar morphology with average diameter of about 8 nm (BaSnO_3_-doped sample) and 6.5 nm (BaZrO_3_-doped sample) and density of 500 *μ*m^−2^ for the both samples. The average length of the BaSnO_3_ nanocolumns is about two times higher than the BaZrO_3_ nanocolumns. The angular dependences of critical current in magnetic field up to 1 Tesla at 77 and 65 K have been obtained. The critical current and its anisotropy depend on artificial pinning centers presence and their type. The angular dependence of resistivity in the field up to 9 Tesla was also studied and discussed.

## Introduction

The commercial production of second generation high temperature superconducting tapes (2G HTS tapes, coated conductors, CC) based on *RE*Ba_2_Cu_3_O_7−*δ*_ (where *RE* is a rare-earth element, *RE*BCO) is rapidly developing in many countries and that can be proved by large number of publications^1–4^. Various devices for power industry based on 2G HTS wires like power cables, fault current limiters, various types of motors and inductive drives etc. have already been designed and tested^5–7^. However, the improvement of CC’s performance in magnetic field is still necessary and that is the goal of a number of investigations. The practice shows that intrinsic defects of CC’s, like point defects, misfit dislocation, twin boundaries, grain boundaries, and other crystal lattice imperfections do not pin vortexes strong enough. One way to increase the in-field stability of CC’s critical current density, *j*_*c*_, at LN_2_ temperatures is the introduction of artificial pining centers (APC). First APC’s in superconducting films were obtained by nano-islands on substrate (substrate decoration) on Ti-base films^8^. The possibility to increase the *j*_*c*_ in YBa_2_Cu_3_O_7−*δ*_ CC’s with APC was shown by MacManus-Driscoll and co-workers in 2004^9^, later on the validity of this approach was demonstrated in a number of papers^10–13^. Introduction of the APC had increased the lift-factor and significantly reduced the critical current (*I*_*c*_) anisotropy in strong magnetic fields (up to 10 Tesla) at *T* ≫ 77 K. The lift-factor is the ratio of *I*_*c*_ at certain temperature and magnetic field value to the *I*_*c*_ of the same sample at 77 K in self-field (s.f.) (lift-factor = *I*_*c*_ (77 K, 1 T, Θ)/*I*_*c*_(77 K, s.f.)). SuperOx company also started the R&D program oriented on high-rate manufacturing of CC’s with perovskite APC^14,15^. It is important to understand the contribution of APCs to the final pinning force value and its dependence on temperature and external magnetic field, including its orientation^16,17^. To solve this problem one have to determine the microstructural characteristics of HTS tapes with a different type of APC. In present study, we compared commercial CC’s, 12 mm wide, without APC (REF sample) and those obtained at the same high rate pulse laser deposition (PLD) of GdBCO layer doped with 6 molar % BaSnO_3_ (BSO sample) and BaZrO_3_ (BZO sample) APC. The correlation of CC’s microstructure studied by transmission electron microscopy (TEM) and their superconducting characteristics was also considered.

## Results

### Phase composition, texture and microstructure of samples

**Figure 1:**
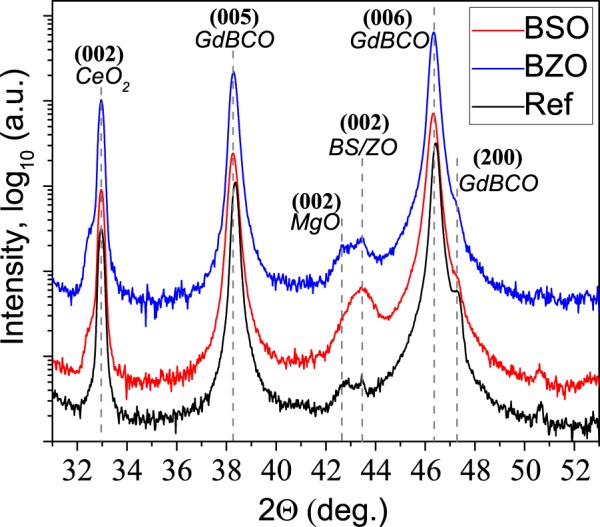
XRD data of the 2G HTS tapes.

The X-Rays diffraction (XRD) data are shown in Fig. 1. The HTS layer and the CeO_2_ top buffer layer have texture with predominant (001) orientations. For the REF sample and BSO sample there is a small amount of (100) oriented grains (*a*-orientation) in the HTSC layer, which is positioned as the right shoulder of (006) GdBCO peak in the XRD scans. Two low-intensity peaks around 44° on the XRD scans of the REF sample correspond to (002) reflection of the MgO buffer layer and (111) reflection of Hastelloy (Ni-based alloy) substrate. More intensive (002) BSO and BZO reflections are also located in this area of the XRD pattern, overlapping with MgO (002) and Ni (111) reflections of the doped samples. Other reflections of APC were not detected and that referred to the texture of APC particles with orientational relationship between APC and HTS as - (001) BSO/BZO‖(001) GdBa_2_Cu_3_O_7−*δ*_.

**Figure 2:**
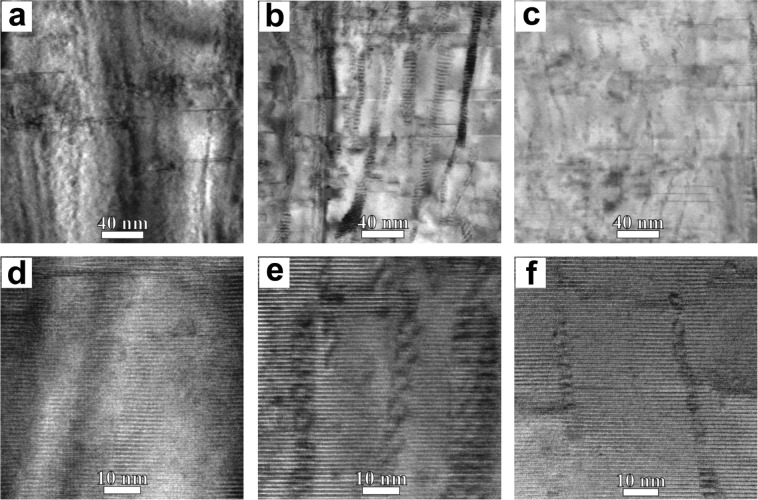
Enlarged TEM cross-section images with different magnifications: (**a**,**d**) reference sample, (**b**,**e**) BSO sample, (**c**,**f**) BZO sample. Enlarged images demonstrates visible APC nanocolumns.

**Figure 3:**
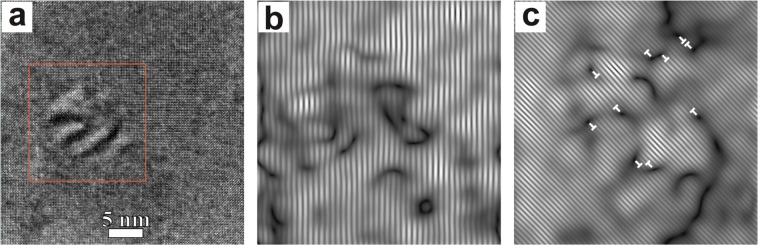
(**a**) HR TEM plan-view image BSO sample and the Fourier filtered images of selected area marked by red square and obtained from the reflexes: (**b**) $0\bar{1}{0}_{GdVCO}$ and 100_*BSO*_, (**c**) $1\bar{1}{0}_{GdBCO}$ and 110_*BSO*_. Extra crystal planes are marked by ⊥.

Bright Field (BF) TEM cross-section overall images of all samples are shown in supplementary Fig. S1. These images demonstrate that the BSO and BZO adopted nanocolumn morphology, with the elongation towards the growth direction of GdBCO layer. The enlarged images of the samples (Fig. 2) demonstrate specific moiré patterns associated with the APC nanocolumns, which arose from the overlay of the APC and HTS matrix crystal lattices. Based on such images the average diameter of nanocolumns and APC density values were calculated and the results are presented in Table 1. We found that the nanocolumns are slightly tilted in relation to the GdBCO *c*-axis. The average tilt angles of the nanocolumns have ≈4.5° and ≈11° for BSO and BZO samples, respectively. The mismatch between the BSO (BZO) and GdBCO crystal lattices can be partly released due to the formation of partial dislocations with the projection of Burgers vector parallel to 110 GdBCO crystal planes, but not to 100 or 010 crystal planes and this is visible on the Fourier filtered images of selected area (Fig. 3 and supplementary Fig. S2). The “1-2-4” plane defects were observed in all the samples and these defects could influence the superconducting properties^16^.

**Table 1:** Electophysical and microstructural characteristics of the 2G HTS tape samples with APC and reference sample.

APC dopant		Undoped sample	BaSnO_3_ sample	BaZrO_3_ sample
*T*_*c*_		93.3	91.9	91.0
*I*_*c*_ at 77 K (A/12 mm)		360	140	120
Density of APC	plan-view estimates (*μ*m^−2^)	—	400 ± 100	500 ± 100
	cross-section estimates (*μ*m^−2^)	—	600 ± 200	600 ± 200
cmAverage diameter of the nanocolumns	plan-view estimates (nm)	—	7.9 ± 3.3	7.1 ± 2.9
	cross-section estimates (nm)	—	8.5 ± 2.2	6.3 ± 1.3

**Figure 4:**
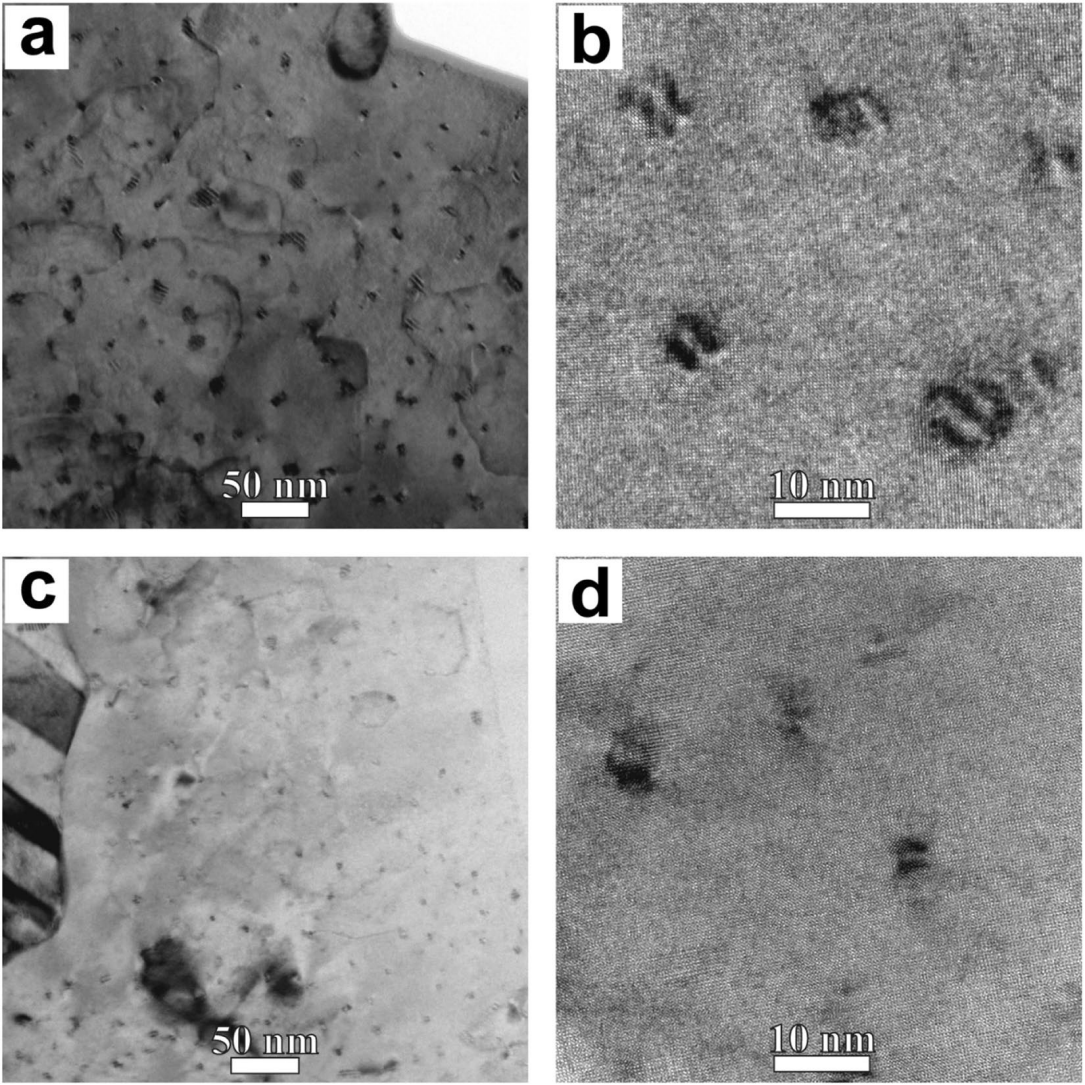
TEM plan-view images with different magnifications (**a**), (**b**) BSO sample; (**c**), (**d**) BZO sample.

Figure 4 shows TEM images of the plan-view samples. The nanocolumns density and their average diameter were determined in these images and the results are presented in Table 1.

### Superconducting properties

**Figure 5:**
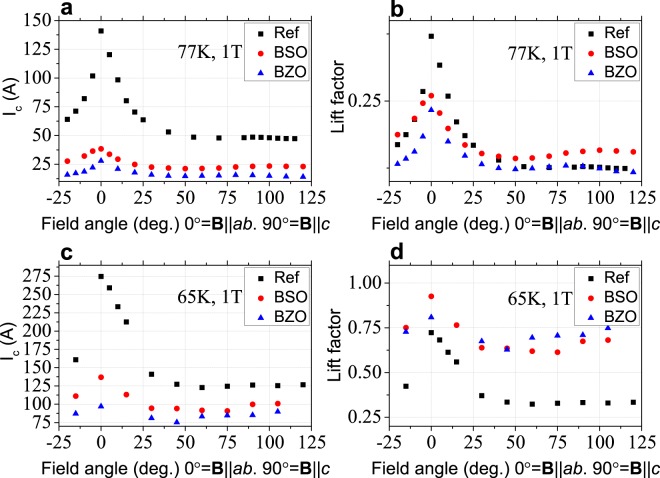
Angular dependence of absolute *I*_*c*_ value (**a**,**c**) and lift-factor (**b**,**d**) at applied magnetic field 1 Tesla, 77 K and 65 K.

The *T*_*c*_ and *I*_*c*_ values of the samples (77 K, s.f.) are presented in Table 1. The angle dependences of critical current for all the samples are presented in Fig. 5. The *I*_*c*_ at 77 K in REF sample is higher than in other samples for all field orientations and *I*_*c*_ in turn is higher in the BSO sample than in BZO sample.

At 65 K the difference of critical currents between reference and doped samples becomes lower. For the field orientation B‖c the *I*_*c*_ values in reference and doped samples become smaller, and *I*_*c*_ is still higher in BSO sample. The lift-factor in the samples with APC is higher at 77 K comparing to REF sample for all field orientations and it reaches the highest value at 65 K. At this temperature and field value of 1 T the angular dependence becomes more isotropic.

### The temperature dependence of the resistivity

The APC introduction resulted in the decrease of *T*_*c*_. The *T*_*c*_ for the REF sample is 93 K. The *T*_*c*_ for the BSO sample and BZO sample are 91.9 K and 91 K, correspondingly (supplementary Fig. S3). The *T*_*c*_ decreases in high magnetic field and the transition broads to few Kelvins in all samples. The infield broadening in BZO sample is less than in REF sample. The larger *T*_*c*_ decrease was observed, when the field orientation was changed from **B**‖*ab* to **B**‖*c*.

**Figure 6:**
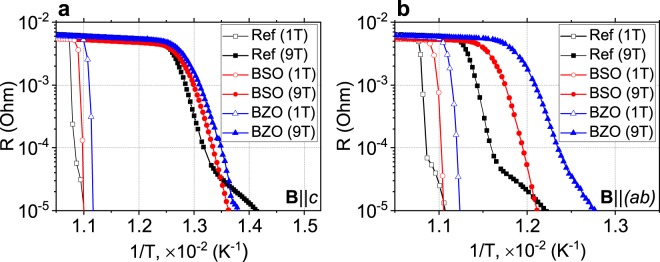
Dependence of In*R* versus 1/T for all samples in the field (**a**) **B**‖*c* and (**b**) **B**‖(*ab*).

The logarithmic of resistivity (In*R*) versus 1/T dependencies are shown in Fig. 6. The In*R* in the REF sample looks differently from the doped samples, with a kink and two ranges of different slopes. At high magnetic field the kink is more evident.

## Discussion

The microstructural analysis of 2 G HTS tapes demonstrates that the BSO and BZO nanocolumns are formed in the doped samples with the typical diameter of ≈8 nm and ≈6.5 nm, respectively. These values are in close correlation with ones found in the previous study^13^. We found that the APCs in both types of samples are not strictly parallel to [001] GdBCO, but were tilted up to 24° (see Fig. 2). Similar results presented in the study of Maeda *et al*.^18^ for BaHfO_3_ embedded in GdBCO matrix. The angular dependence of the nanocolumns on the substrate temperature and growth rate were discussed by Ichino *et al*.^19^. The APC insertion leads to a drop of *T*_*c*_. The authors of next article indicated that *T*_*c*_ is influenced by uniaxial stress and we suppose that the strains at the B(S/Z)O/GdBCO interface could cause the change of *T*_*c*_^20^. The cooling down to 65 K increases the critical current value and lift-factor in comparison with the critical current value and lift-factor at 77 K and also decreases its anisotropy under the external magnetic field, this is clearly observed in the angular dependencies of the critical current in the magnetic fields up to 1 T. These data demonstrate the efficiency of APC. The *I*_*c*_ values and the lift-factor increase even more with further decrease of temperature and further increase of the external fields up to 9 T, which was demonstrated in our previous publications^14,15^.

External magnetic field shifts down and enlarge the temperature range of transition to superconducting state. The In*R* vs 1/T graphs reveal two ranges of different slopes in the vicinity of *T*_*c*_, the difference is more expressed when the field is increased up to 9 T (Fig. 6). The observation is accompanied by the fact that the magnetic field influenced differently on the samples when it was turned from **B**‖*c* to **B**‖(*ab*). These results indicate together that two types of pinning centers exist in the REF GdBCO sample which can be nanoparticles Gd_2_O_3_ and planar defects containing extra copper oxide easy detectable by TEM. This conclusion correlates also with the results presented in recent publication^21^. In the field **B**‖(*ab*), evidently, the planar defects are mostly effective. In the sample with the APC, the predominant contribution of transverse type defects can be explained by the fact that the APC size is larger than the size of the plane defects and comparable to the size of the vortexes. Thus vortexes pin preferably on these APC defects.

The external magnetic field reduces the activation energy for all types of samples. For the REF sample and the BSO sample, the activation energy in the 1 T field **B**‖*c* is of ≈0.95 eV, and for the BZO sample is of 0.86 eV. In the field **B**‖(*ab*), the activation energy for all types of samples increases slightly as compared with the case, when **B**‖*c*. As the external magnetic field increases, the activation energy also increases. It is worth noting that the rotation of the external magnetic field leads to a decrease in the activation energy for all samples. This decrease is larger for the REF and BSO samples.

The close inspection of cross-section images of whole HTS layers shows that APC average length (*L*_*a*_) of the nanocolumns in the BSO sample is described by a log-normal distribution log *N* (*μ*, *σ*^2^) with parameters *μ* = 4.56 and *σ* = 0.48. The *L*_*a*_ value (determines as expectation) is 107.1 nm. For the BZO sample the log *N* (*μ*, *σ*^2^) distribution has the parameters *μ* = 3.67 and *σ* = 0.58, the value of *L*_*a*_ is 46.5 nm. Thus, it was found, that *L*_*a*_ value of the nanocolumns is about twice larger in the BSO sample. There is a good match of these data with the field dependence of pinning force, (supplementary Fig. S4), and the results of recent study^22^. The authors of that study concluded that a higher value of current density corresponds to the longer mean length and a smaller average diameter of the APC. The value of the pinning force for our BSO sample and BZO sample are 32.1 N · m^−3^ and 22.5 N · m^−3^, respectively. We followed the postulates of the authors^22^ for the estimation of the pinning energy: *U* = *L*_*a*_ × *u*_0_ and authors^23^ for the calculation of pinning energy per unit length: ${u}_{o}=\frac{1}{2}{\varepsilon }_{0}\,ln(1+{(\frac{{C}_{0}}{\sqrt{2}{\xi }_{ab}})}^{2})$, where: ${\varepsilon }_{0}={({\Phi }_{0}/4\pi {\lambda }_{ab})}^{2}$, *C*_0_ — radius of APC, Φ_0_ — flux quantum, ${\lambda }_{ab}={\lambda }_{0}\,{(1-{t}^{4})}^{-0.5}$ and ${\xi }_{ab}={\xi }_{0}\,{(1-t)}^{-0.5}$, where *t* = *T*/*T*_*c*_. Taking into account the mean length estimated by the TEM, the pinning energy for BSO sample and BZO sample are $U={L}_{a}\times {u}_{0}=0.85\times {10}^{-19}\,J=0.91$ eV and $U={L}_{a}\times {u}_{0}=0.12\times {10}^{-19}J$ = 0.59 eV, correspondingly. The difference between these values is ≈1.6 times due to the larger average length of the APC in BSO sample. In this sample, the nanocolumns produce more valued effect on the anisotropy reduction and do not suppress strongly *T*_*c*_ value in comparison with the REF sample.

## Methods

The GdBCO layers on IBAD MgO substrates with CeO_2_ terminating layer were formed by PLD in reel-to-reel regime with layer growth rate of 750 nm · min^−1^, which is typical for the pilot-scale equipment at SuperOx. The samples preparation in more details was described in^14,15^. The schematic representation of the sample microstructure is shown in supplementary Fig. S5.

The XRD was performed in parallel beam geometry in a 5-circle Rigaku SmartLab diffractometer (Rigaku Corp., Japan) equipped with a 9 kW X-ray source with rotating copper anode. 2Θ/*ω*-scan was performed for the phase analysis and *ω*- and *j*-scans aimed texture sharpness analysis.

TEM studies were performed on a S/TEM Titan 80–300 (Thermo Fisher Scientific, USA) equipped with a spherical aberration probe corrector, an energy dispersive X-ray spectrometer (EDAX, USA) and high angle annular dark field detector (Fischione, USA). The microscope was operated at 300 kV. The JEMS software developed by P. Stadelman was used for electron diffraction patterns and image simulations^24^. Cross-sectional samples were prepared using a focused Ga^+^ ion beam in a scanning electron microscope (FIB/SEMs) Helios Nanolab 600i (Thermo Fisher Scientific, USA) equipped with a Pt, W gas injection systems (GIS) and an Omniprobe 200 micromanipulator (Omniprobe, USA). To obtain planar-sections of the samples, a FIB/SEMs Versa 3D DualBeam (Thermo Fisher Scientific, USA), equipped with a Pt and W GIS, and an EasyLift micromanipulator (Thermo Fisher Scientific, USA) was used in high vacuum mode.

Angular dependences of the *I*_*c*_ at 65 and 77 K in the fields up to 1 T were measured by four-probe technique described in^17^. Other electrophysical measurements and the temperature dependence of the resistivity were carried out using PPMS-9 (Quantum design, USA). The following experiment parameters were used in the four-probe measurements: the distance between the contacts was of 6.5 mm and DC-current — 100 mA. The magnetic field was varied between 0 and 9 T (with exact values 0.1, 0.3, 1, 3, and 9 T).

## Supplementary information

Supplementary Information
