# Supplementary material for: Microstructure and superconducting properties of high-rate PLD-derived GdBa2Cu3O7−δ coated conductors with BaSnO3 and BaZrO3 pinning centers
Source: Sci Rep. 2019 Oct 23;9:15235. doi: 10.1038/s41598-019-51348-w (PMC6811553; doi:10.1038/s41598-019-51348-w)
Supplement: Supplementary file 1 — Supplementary Information [file 41598_2019_51348_MOESM1_ESM.pdf]

Microstructure and superconducting properties of high-rate PLD-derived $GdBa_2Cu_3O_{7-\delta}$ coated conductors with $BaSnO_3$ and $BaZrO_3$ pinning centers

Alexey V. Ovcharov[1], Pavel N. Degtyarenko[2,3], Vsevolod N. Chepikov[2,4], Alexander L. Vasiliev[1,5,6,*], Sergey Yu. Gavrilkin[7], Igor A. Karateev[1], Alexey Yu. Tsvetkov[7], and Andrey R. Kaul[2,4]

[1]National Research Center ”Kurchatov Institute”, Moscow, 123182, Russia
[2]SuperOx, Moscow, 117246, Russia
[3]Joint Institute for High Temperature of Russian Academy of Sciences, Moscow, 125412, Russia
[4]Lomonosov Moscow State University, Moscow, 119991, Russia
[5]Shubnikov Institute of Crystallography of Russian Academy of Sciences, Moscow, 117333, Russia
[6]Moscow Institute of Physics and Technology (State University), Dolgoprudny, Moscow Region, 141701, Russia
[7]P.N. Lebedev Physical Institute of the Russian Academy of Sciences, Moscow, 119991, Russia
[*]a.vasiliev56@gmail.com

Content:

Figure S1. TEM cross-section overall images of samples.

Figure S2. HR TEM plan-view image of the BSO sample and the Fourier filtered images.

Figure S3. Temperature dependence of resistivity for reference and doped samples.

Figure S4. Field dependence of pinning force for doped samples in field **B**||*c* at 77 K.

Figure S5. Schematic layer-by-layer architecture of the samples.

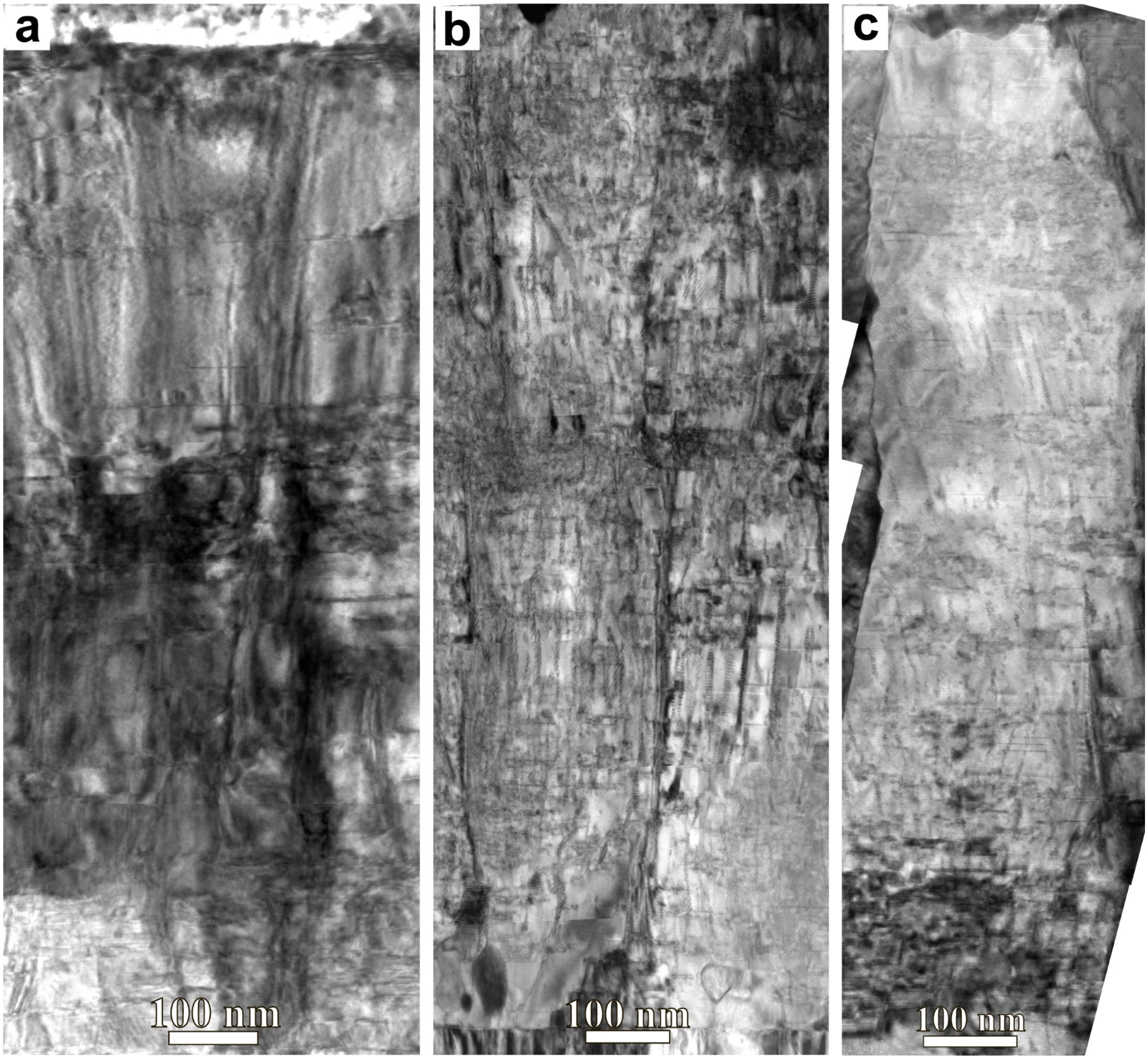

Figure S1. TEM cross-section overall images: (**a**) reference sample, (**b**) 6% BSO sample, (**c**) 6% BZO sample.

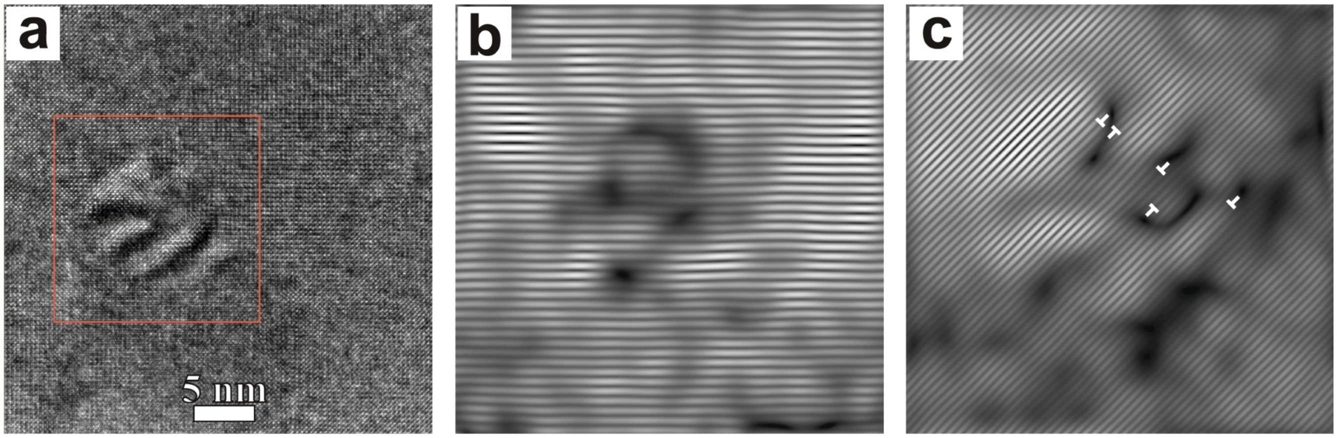

Figure S2. (**a**) HR TEM plan-view image of the BSO sample and the Fourier filtered images of selected area marked by red square and obtained from the reflexes: (**b**) 100_{GdBCO} and 010_{BSO}, (**c**) 110_{GdBCO} and $\bar{1}10_{BSO}$. Extra crystal planes are marked by ⊥.

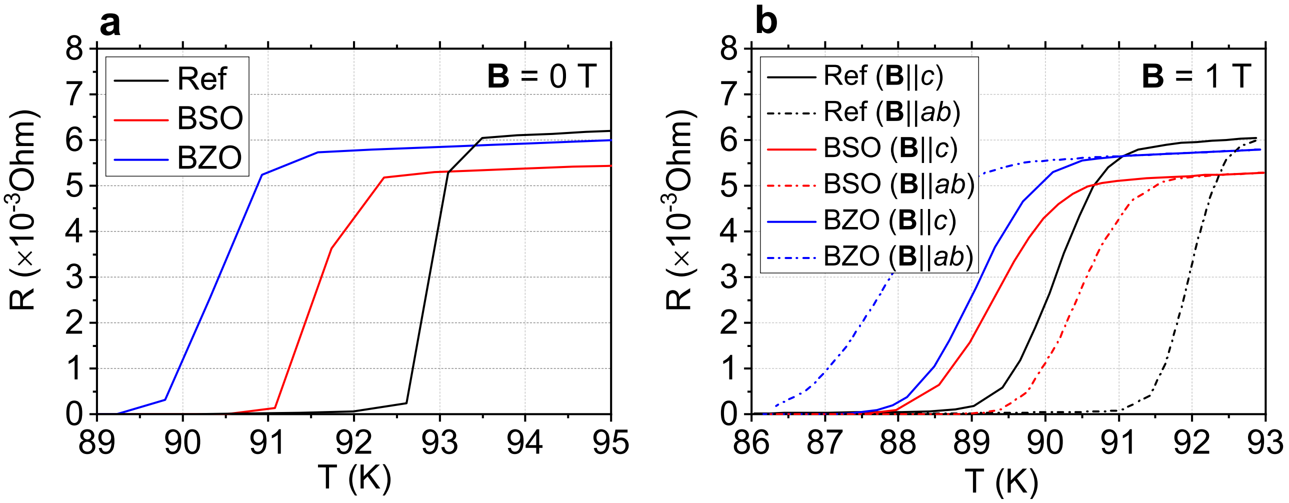

Figure S3. Temperature dependence of resistivity for reference and doped samples. (**a**) – without applied magnetic field. (**b**) – at 1 T **B**||*c* – solid curves, **B**||*(ab)* – dash curves.

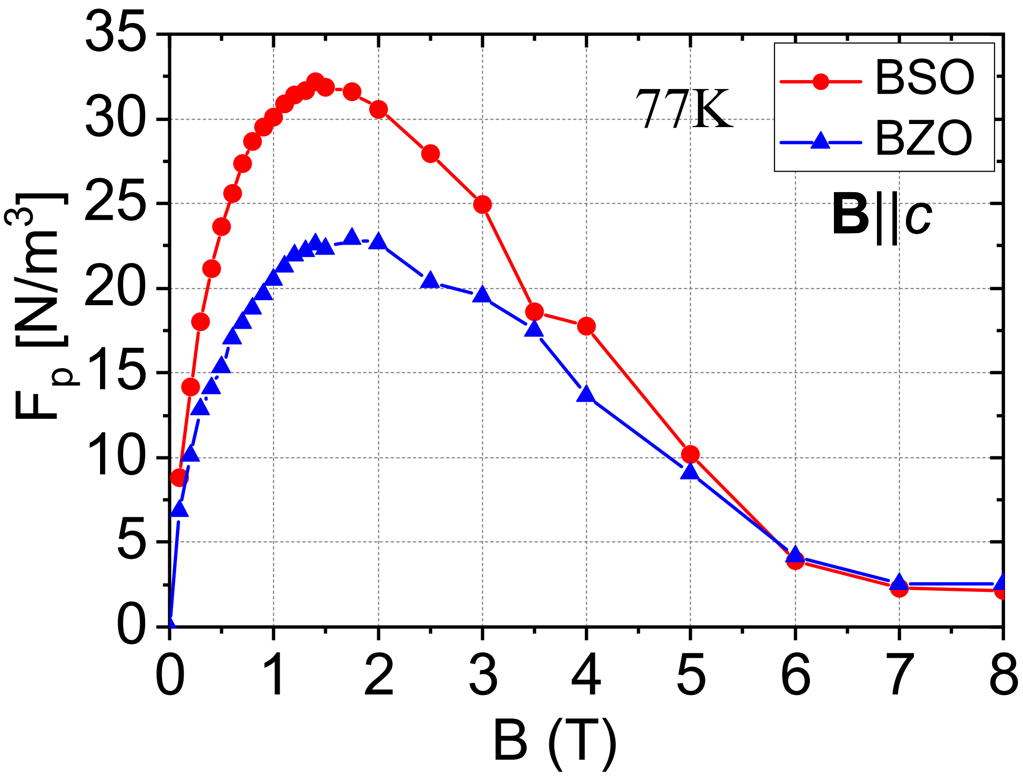

Figure S4. Field dependence of pinning force for doped samples in field **B**||*c* at 77 K.

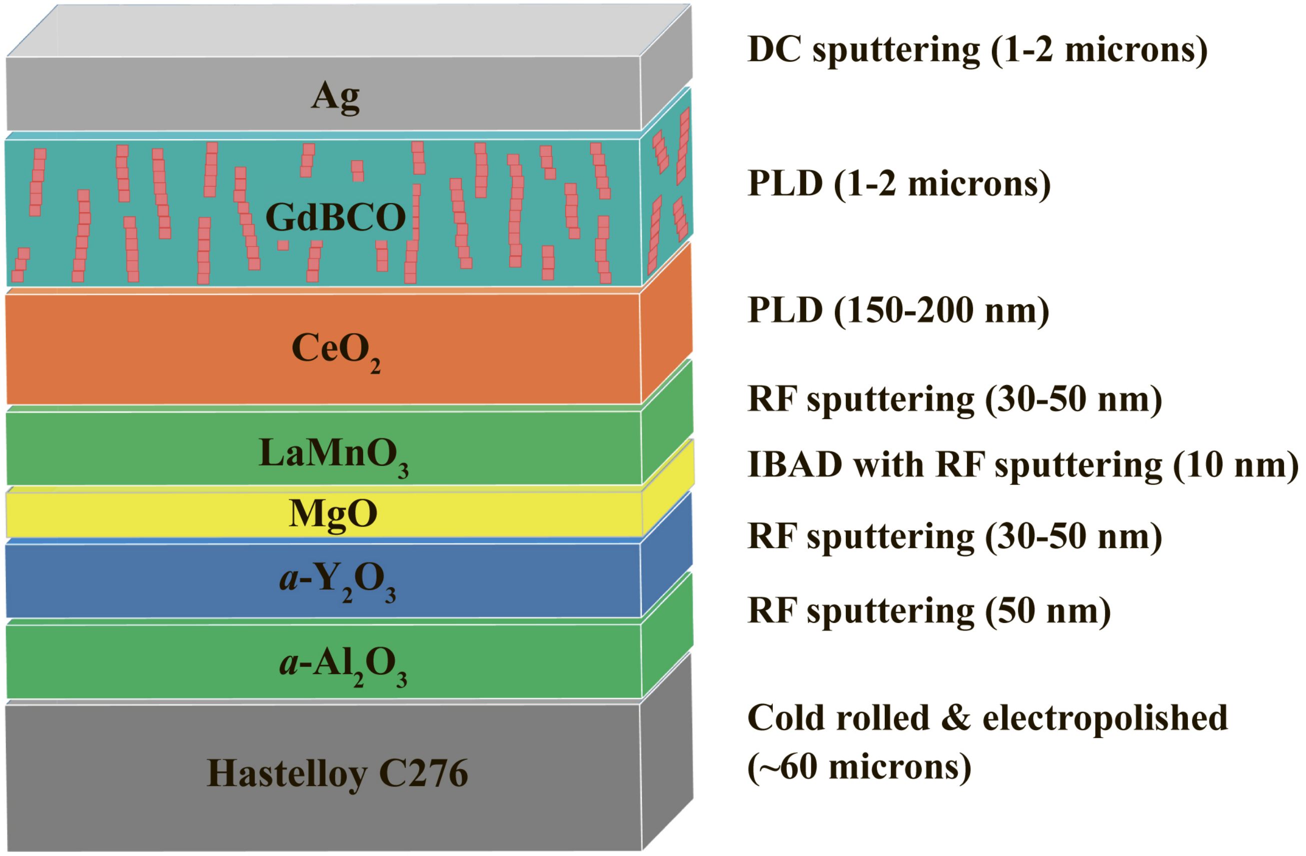

Figure S5. Schematic layer-by-layer architecture of the samples. The superconducting layer contains artificial pinning centers that marked as coral nanocolumns.
